# Structured medication reviews: origins, implementation, evidence, and prospects

**DOI:** 10.3399/bjgp21X716465

**Published:** 2021-07-30

**Authors:** Duncan Stewart, Mary Madden, Paul Davies, Cate Whittlesea, Jim McCambridge

**Affiliations:** Centre for Primary Health and Social Care, London Metropolitan University, London.; Department of Health Sciences, University of York, York.; Stephenson Park Health Group, Newcastle upon Tyne.; University College London (UCL) School of Pharmacy, UCL, London.; Department of Health Sciences, University of York, York.

Pharmacists have been employed in UK general practice for many years. Their numbers are now expanding and their roles developing. Clinical pharmacists are expected to alleviate workload pressures on GPs. Notwithstanding the COVID-19 vaccination programme, a new Structured Medication Review (SMR) service has been introduced in Primary Care Networks (PCNs). The long term drivers are clear: addressing problematic polypharmacy in the NHS, reducing avoidable hospitalisations, and delivering better value from medicines spending.^1^ SMRs are intended to improve the quality of prescribing, delivering improvements to patient care and outcomes.

## RAPID IMPLEMENTATION OF PCNs AND THE SMR

The roll-out of PCNs and the development of the SMR specification were both done at speed. Collaboration between GP practices to form PCNs under the Network Contract Directed Enhanced Service (DES) requires time and goodwill to build the relationships needed to manage clinical, organisational, and business interests collectively.^2^ Short timescales between policy statements, guidance, and expected implementation have jeopardised effective planning, adding to pressures on clinicians and services during the COVID-19 pandemic.

SMRs were one of the first five DES specifications proposed, originally to be delivered to a range of patient priority groups from April 2020. In response to initial consultation, additional new PCN roles were added, including pharmacy technicians. The proposed SMR service was also simplified by reducing the number of target populations and allowing the volume of SMRs to be determined locally.^3^ COVID-19 planning then took centre stage and has interrupted implementation profoundly. Guidance issued in March 2020 stressed prioritising responses to the pandemic, thus delaying introduction of the SMR. Vaccination was identified as a workforce priority in early 2021.^4^

The full SMR specification, first published shortly before its formal introduction in September 2020, included the wider offer of SMRs to patients who might benefit, subject to clinical pharmacist capacity.^5^ New information (in an annex) expanded the scope of SMRs to include attention to the main public health behavioural risk factors of smoking, physical activity, diet and weight management, and alcohol, as well as falls prevention.^5^ Further amendments have been made since, including expanding the range of targeted potentially addictive medicines, specifying a minimum consultation duration of 30 minutes, and bringing children into scope.^6^

Important changes with implications for the developing PCN clinical pharmacist role have thus been made at short notice, and a time when much of the NHS workforce has been focused on managing the pandemic. This raises questions about expectations, preparedness, and practice development.^7^ SMR conduct is to be personalised, holistic, and conducted in line with the principles of shared decision making, attentive to health literacy, and likely to be largely delivered remotely for now.^6^ Newly recruited clinical pharmacists conducting SMRs are required to have, or be in training for, a prescribing qualification, and to have advanced assessment and history-taking skills.^6^ Many are coming from the community pharmacy workforce where such skills are not a requirement. Since an initial 18-month training pathway (or equivalent) must first be completed, the SMR is being introduced when few are fully trained, and when training and supervision provision has been impacted by the pandemic.^7^

In socioeconomically deprived areas where polypharmacy is more common and there are underserved populations, the SMR could represent an especially important innovation. Little information is provided in the DES that appreciates the nature of any distinct challenges posed, or how they may be addressed. This is for each PCN to determine. Similarly, content on how to incorporate brief advice for public health purposes in ways underpinned by shared decision-making principles is limited. In the case of alcohol, for example, alongside advice-giving, the annex notes the potential for interactions with medicines and the need for review, neither of which are congruent with the recommended form of brief advice.

There is further work to be done in developing the service and monitoring implementation if SMRs are to make the kinds of contribution that are anticipated. Account must also be taken of what is known about the nature of the person-centred consultation skills possessed by the new clinical pharmacist workforce within primary care, and the strengths and limitations of the existing evidence.

## SMR EVIDENCE

Pharmacist-led medicine reviews have an important role to play in improving prescribing safety.^8^ The original design of the SMR service and the subsequently made amendments have, however, engaged with research evidence in limited ways. National Institute for Health and Care Excellence (NICE) clinical guidelines for medicines optimisation^9^ are cited as the principal source of evidence for SMRs.^5^^,^^6^ Closer examination shows weaknesses in this evidence base. Most trials reviewed by NICE were of low to moderate quality and reported mixed findings for diverse outcomes in populations that do not resemble those identified as priorities for the SMR.^9^

There is a growing appreciation of the potential contribution of the expanded pharmacist role in primary care,^10^^,^^11^ and established GP practice pharmacists’ experience may facilitate SMR implementation in some practices. However, few studies investigate actual practice, such as how pharmacy work is routinely conducted,^10^ nor is there evidence to show that pharmacists possess skills in shared decision making as operationalised in general practice. Studies in community pharmacy and primary care identify a substantial gap between patient-centred consultation ideas encouraging patient-generated problem solving and medicines review practice centred on information provision.^12^^,^^13^ In general practice, both GPs and pharmacists perceive a trade-off between being time efficient and involving the patient in making decisions about their medicines.^14^

Incorporating public health brief advice generates questions about how the targets (for example, obesity) may be implicated in issues raised in medicines-focused work, such as in relation to adherence. Where it exists, available evidence supportive of pharmacists addressing these as standalone issues (for example, as adjuncts) is, at best, highly variable.^15^ Alcohol provides another instructive example. The SMR specification highlights the risks to patient safety from interactions with medicines.^16^ Recognition of the far reaching nature of the issues with which alcohol is implicated^17^ is a welcome first step in advancing thinking about clinical approaches for both prevention and treatment of multimorbidity.^18^ Implicit in the SMR is a more advanced conception of prevention for alcohol than the narrow *NHS Long Term Plan* focus on dependent drinkers and averting their hospitalisation.^19^ This could usefully become more explicit. Conceptualising alcohol as a drug^20^ fits with the core pharmaceutical role, meaning pharmacists may regard the SMR as a legitimate and important venue for addressing alcohol use in routine medicine consultations. Doing so could contribute to a strategic shift in how the NHS thinks about and manages the relationships between such behavioural risk factors, chronic conditions, and care provided. It potentially offers a smarter way forward than promoting widespread dissemination of crude advice, with disappointing uptake.^21^

## THE FUTURE OF SMRs

The challenges facing the new clinical pharmacist workforce were already formidable, and have been made more so by the COVID-19 pandemic.^2^ The balance of forces between centralised contractual implementation and locally tailored innovations will play out in different ways in different places. The active involvement of pharmacists in taking on clearer leadership roles in medicines review practice developments is paramount for the potential of SMRs to be realised. This requires ongoing evaluation of how pharmacists develop their emerging roles in primary care and acquire more advanced person-centred clinical skills in delivering SMRs for diverse and complex needs. General practice and the quality of the service received by patients can benefit, with patients helped to use their medicines in ways that work better for them, improving population health as a result.

## References

[1] NHS England, NHS Improvement (2019). Network Contract Direct Enhanced Service: draft outline service specifications.

[2] Pettigrew LM, Kumpunen S, Mays N (2020). Primary care networks: the impact of covid-19 and the challenges ahead. BMJ.

[3] NHS England, NHS Improvement (2020). Network Contract Directed Enhanced Service: contract specification 2020/21 — PCN requirements and entitlements.

[4] NHS England, NHS Improvement (2021). Supporting general practice in 2021/22.

[5] NHS England (2020). Network Contract Directed Enhanced Service Structured medication reviews and medicines optimisation: guidance.

[6] NHS England (2021). Network Contract Directed Enhanced Service Structured medication reviews and medicines optimisation: guidance.

[7] Royal Pharmaceutical Society (2020). Network Contract Direct Enhanced Service: draft outline service specifications Royal Pharmaceutical Society response.

[8] Clyne B, Fitzgerald C, Quinlan A (2016). Interventions to address potentially inappropriate prescribing in community-dwelling older adults: a systematic review of randomized controlled trials. J Am Geriatr Soc.

[9] National Institute for Health and Care Excellence (2015). Medicines optimisation: the safe and effective use of medicines to enable the best possible outcomes.

[10] Anderson C, Zhan K, Boyd M, Mann C (2019). The role of pharmacists in general practice: a realist review. Res Social Adm Pharm.

[11] Bradley F, Seston E, Mannall C, Cutts C (2018). Evolution of the general practice pharmacist’s role in England: a longitudinal study. Br J Gen Pract.

[12] Latif A, Boardman HF, Pollock K (2013). Understanding the patient perspective of the English community pharmacy Medicines Use Review (MUR). Res Social Adm Pharm.

[13] Morris S, Madden M, Gough B (2019). Missing in action: insights from an exploratory ethnographic observation study of alcohol in everyday UK community pharmacy practice. Drug Alcohol Rev.

[14] Duncan P, Cabral C, McCahon D (2019). Efficiency versus thoroughness in medication review: a qualitative interview study in UK primary care. Br J Gen Pract.

[15] Brown TJ, Todd A, O’Malley C (2016). Community pharmacy-delivered interventions for public health priorities: a systematic review of interventions for alcohol reduction, smoking cessation and weight management, including meta-analysis for smoking cessation. BMJ Open.

[16] Holton AE, Gallagher PJ, Ryan C (2017). Consensus validation of the POSAMINO (POtentially Serious Alcohol Medication INteractions in Older adults) criteria. BMJ Open.

[17] McCambridge J, Stewart D (2020). Managing alcohol use in primary care. BMJ.

[18] Stewart D, McCambridge J (2019). Alcohol complicates multimorbidity in older adults. BMJ.

[19] NHS England (2019). The NHS Long Term Plan.

[20] Kypri K, McCambridge J (2018). Alcohol must be recognised as a drug. BMJ.

[21] McCambridge J, Saitz R (2017). Rethinking brief interventions for alcohol in general practice. BMJ.

